# Palliation with Oesophageal Metal Stent of Pseudoachalasia from Gastric Carcinoma at the Cardia: A Case Report

**DOI:** 10.1155/2009/791627

**Published:** 2009-09-06

**Authors:** Salvatore Maria Antonio Campo, Roberto Lorenzetti, Marina de Matthaeis, Cesare Hassan, Angelo Zullo, Paola Cerro, Sergio Morini

**Affiliations:** ^1^Gastroenterology Unit, Nuovo Regina Margherita Hospital, Via Morosini 30, 00153 Rome, Italy; ^2^Radiology Unit, Nuovo Regina Margherita Hospital, Via Morosini 30, 00153 Rome, Italy

## Abstract

We present an 82-year-old woman with a 3-month history of progressive dysphagia and a normal initial upper gastrointestinal endoscopy. The diagnosis of pseudoachalasia was suspected by oesophageal manometric and barium swallow studies, and confirmed by biopsies revealing an intestinal type carcinoma of the stomach at a repeated endoscopy. In view of the history of heart disease, diabetes, and old age, this patient was treated by a partially covered Ultraflex self-expanding metal stent (Boston Scientific, Natick, MA, USA) placed into the oesophageal body with no direct complications and obtaining the relief from dysphagia. During the 11-month follow-up she was treated for an iron deficiency anaemia due to reflux oesophagitis with ulcerations in the oesophageal body and died from myocardial infarction. According to the localization of the cancer, the old age, and the presence of comorbidities, we should recommend the insertion of a partially covered self-expanding metal stent as a reasonable palliative treatment in selected subjects with pseudoachalasia.

## 1. Introduction

Pseudoachalasia was firstly recognized by Ogilvie in 1947 due to the involvement of the distal oesophagus, with submucosal infiltration of the lower oesophageal sphincter (LES) and cardia by a carcinoma as the most usual cause [1]. Several reports have considered pseudoachalasia as a secondary form of achalasia showing that these patients, in contrast to those with idiopathic achalasia, are more likely to be older (>60 years), with short duration of symptoms (<1 year), and presenting with substantial weight loss [2–6]. It mimics idiopathic achalasia because of the same manometric findings, that is, loss of peristalsis in the oesophageal body and lack of relaxation of the LES [7–11]. The most common pathogenetic mechanism is the direct involvement of the oesophageal myenteric plexus by neoplastic cells from a wide variety of malignant cancers infiltrating the mucosa at the cardia, although a distant neoplasm may be seldom the cause [12, 13]. Pseudoachalasia may be difficult to diagnose in the early phase because of the low diagnostic yield of either barium and endoscopic examinations, false-negative rate up to 25% for endoscopic biopsies to diagnose cancer being reported [14].

## 2. Case Report

An 82-year-old white woman was referred to our Unit with a 3-month history of dysphagia initially for solids, then also for liquids, and weight loss. She was diagnosed with diabetes and heart failure with arrhythmia because of a mitral valve insufficiency 10 years before. An upper endoscopy performed elsewhere 3 months before showed a normal oesophagus and gastrooesophageal junction. She was unsuccessfully treated with a 1-month course of proton pump inhibitors (PPIs) because of the suspicion of a nonerosive reflux disease. To rule out the diagnosis of achalasia we initially performed an oesophageal manometry, which showed loss of peristalsis in the oesophageal body, but it failed to investigate the LES function because the manometric catheter could not be passed across the cardia. Thereafter, a barium swallow study found a dilated oesophagus containing some food residue with an irregular tapered narrow cardia. At this point a new upper endoscopy confirmed a dilated oesophagus containing food. Moreover, by pushing hard the scope through the narrow cardia, a mild irregularity of the cardia mucosa was evident. Biopsies revealed an intestinal cell type carcinoma. To assess the extent of the cancer, a CT scan was performed, disclosing only the thickening of the gastric wall confined to the cardia without evidence of metastasis. 

## 3. Patient's Outcome, Current Treatment

Neither surgical treatment nor radiotherapy or chemotherapy were considered suitable in such a patient with a long history of diabetes and heart disease, whereas the insertion of self-expanding metal stent was optioned as a low risk procedure to rapidly improve her quality of life. We chose the Ultraflex self-expanding midsection covered distally releasing oesophageal metal stent (Boston Scientific, Natick, MA, USA) because it provided the most favourable results as far as stent placement, migration, long-term relief of dysphagia, and occurrence of fewer complications [15–18]. 

**Figure 1:**
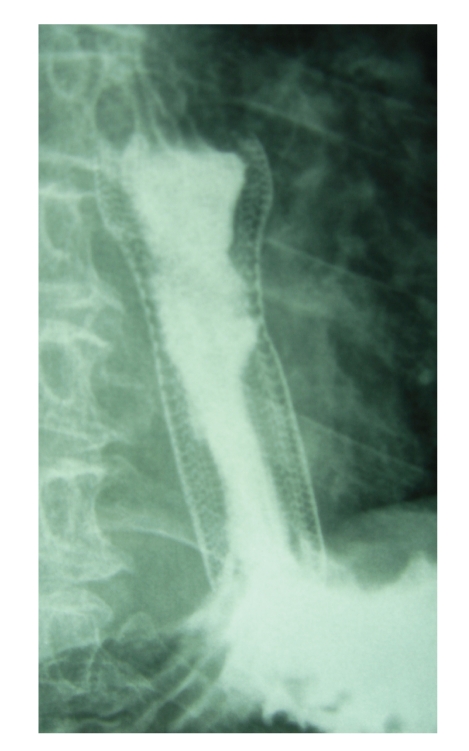
Contrast study shows the well-positioned stent into the oesophageal body.

**Figure 2:**
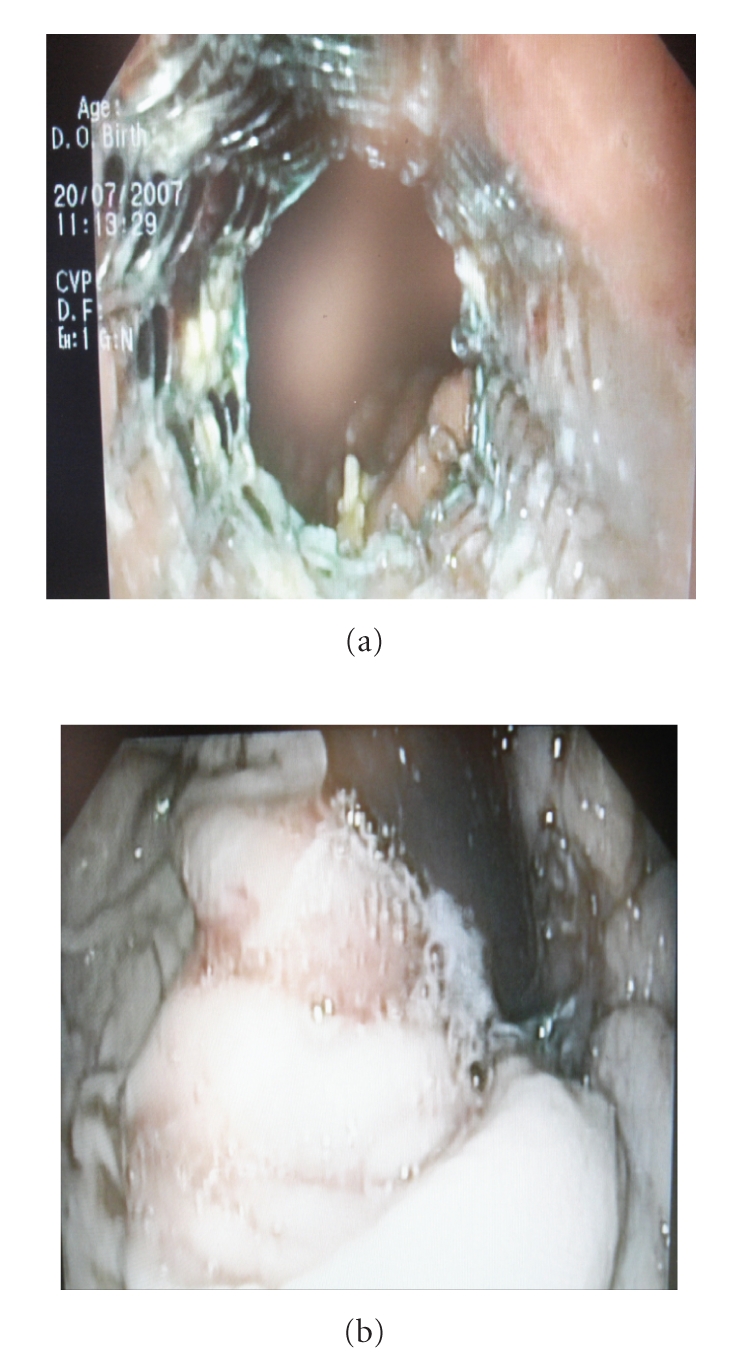
Endoscopic study shows the well-placed stent in the oesophagus without evidence of cancer at the cardia.

The stent had a length of 7 cm, with an 18 mm diameter of the body and its flared proximal end 23 mm, and consisted of a knitted nitinol wire tube with a polyurethane layer, which covered the midsection of the stent, and it was distally released under fluoroscopic control (Figure 1). The proximal flare of 23 mm was able to prevent the most common complication, that is, occurrence of stent migration, while the body diameter of 18 mm seemed to fit appropriately with the dilated oesophagus of a patient with pseudoachalasia. Endoscopic assessment showed the correct oesophageal placed stent without apparent evidence of neoplasia (Figures 2(a)  and 2(b)). In the short period, she was able to eat without complaining dysphagia and she received omeprazole therapy. On the long term she presented iron deficiency anaemia, which was treated with iron supplementation and blood transfusions, and she was recommended to sleep in a sitting position to avoid regurgitation. The patients' quality of life remains acceptable and quite stable during the follow up. An upper endoscopy was done only 3 months later showing that the cause of anemia was the ulcerations in the oesophageal body either as a complication of stent placement or a reflux oesophagitis. The oesophageal stent was correctly placed without migration or tumor overgrowth. She was followed up by telephone interviews for other 9 months when she died because of a myocardial infarction.

## 4. Discussion

Pseudoachalasia is a rare entity, which has only been described in a small subgroup of patients (5%) with two patterns of malignant tumor infiltrating the mucosa at the cardia. The most common type consists of malignant stricture of the cardia which acts as a physical barrier to the passage of food. A less frequent type is strictly related to the malignant submucosal infiltration with secondary impairment of oesophageal postganglionic LES innervations, maintaining the manometric pattern of achalasia stable even after treatment [19]. 

Pseudoachalasia may be difficult to diagnose because multiple diagnostic procedures may fail to indicate the neoplastic origin [14]. In particular, the findings of normal biopsy or negative CT scans results should not lead to complete reassurance of a benign aetiology, that may be achieved only after repeated studies or surgical exploration [20, 21]. Oesophageal endoscopic ultrasound can provide the level of tumor invasion, and the possible spread to regional lymph nodes, but it has low accuracy in differentiating mucosal from submucosal lesions at the lower oesophagus or gastrooesophageal junction [22]. Oesophageal manometry is important for the correct diagnosis in conjunction with carefully barium swallow study, accurate endoscopic examination and biopsy of any mucosa irregularity at the cardia region [2, 20].

In the pseudoachalasia the removal of the obstruction at the cardia by either surgery and/or chemotherapy and/or radiation may often allow the return of normal peristalsis into the oesophagus [23]. However, in a minority of patients with pseudoachalasia, where histological examination of the distal oesophagus has shown infiltration of the myenteric plexus, the typical oesophageal motor disorder has been found stable even after a radical treatment of the neoplasia [12, 13]. In these cases the rapidly relief of the dysphagia and an acceptable quality of life without other more invasive or debilitating treatments may be considered as the main goal in view of their short-life expectancy. 

Recently, the use of expandable oesophageal metal stents have been proposed as additional therapeutic option in achalasia and in a few cases of pseudoachalasia when the other palliative options, that is, oesophageal dilation or surgery or botox, failed. Moreover, temporary use of self-expanding metal stents have been used for benign oesophageal stenosis and fistula in patients who failed other conventional treatments [24–27]. Despite the initial therapeutic success, the overall results were found to be disappointing, frequent complications, such as aorta-enteric fistula, oesophageal perforation, stent migration, and severe reflux oesophagitis being reported [28, 29]. In detail, an acceptable success has been achieved only in those patients with either malignant or benign stricture of cardia, including achalasia, by positioning a permanent or temporary expandable oesophageal metal stent. Unfortunately, neither details on stent characteristics nor full clinical information have been provided in these cases [27, 28]. 

As far as the use of oesophageal metal stents in pseudoachalasia is concerned, data are scanty and basically disappointing (Table 1) [25]. Based on peculiar clinical characteristics of our patient, we attempted to place an oesophageal self-expanding partially covered metal stent, either pneumatic dilation or botox injection at the LES having a high probability of failure. Moreover, pneumatic dilation is associated with a definite risk of perforation. We achieved a therapeutic success with a good quality of life, anemia due to erosive oesophagitis with regurgitation being the only complications. 

In conclusion, the insertion of a self-expanding metal stent into the oesophageal body should be proposed in particular types of pseudoachalasia caused by a cancer at the cardia when a short-life expectancy is presumed or other therapeutic approaches are impracticable. 

## Figures and Tables

**Table 1:** Clinical course of patients with expandable metal stents for achalasia or pseudoachalasia (from reference 25, modified).

Age	Sex	Diagnosis	Dilation	Botox	Metal Stent	Duration	Follow-up/death
86	M	acha	x6	not done	Wallstent I	2 days	proximal stent migration
80	M	acha	x4	x2	Wallstent I	3 months	stent stenosis
81	F	acha	x6	not done	Wallstent I	6 months	stent occlusion by food bolus
81	F	acha	x2	x2	Wallstent II	2 weeks	proximal stent occlusion
31	F	pseudoacha	x3	x2	Gianturco Rosch	slipped	oesophageal cancer metastasis/death
81	F	pseudoacha	x3	x2	Wallstent II	2 weeks	myocardial infarction/death
